# Characterisation of left ventricular function by tissue Doppler imaging technique in newly diagnosed, untreated hypertensive subjects

**Published:** 2008-10

**Authors:** Adedeji K Adebayo, Okechukwu S Ogah, Dike B Ojji, Moshood A Adeoye, Kenneth C Ochulor, Evbu O Enakpene, Olulola O Oladapo, Adewole A Adebiyi, Ayodele O Falase, Olayinka O Ogunleye

**Affiliations:** Division of Cardiovascular Medicine, Department of Medicine, University College Hospital, Ibadan, Nigeria; Division of Cardiovascular Medicine, Department of Medicine, University College Hospital, Ibadan, Nigeria; Division of Cardiovascular Medicine, Department of Medicine, University College Hospital, Ibadan, Nigeria; Division of Cardiovascular Medicine, Department of Medicine, University College Hospital, Ibadan, Nigeria; Division of Cardiovascular Medicine, Department of Medicine, University College Hospital, Ibadan, Nigeria; Division of Cardiovascular Medicine, Department of Medicine, University College Hospital, Ibadan, Nigeria; Division of Cardiovascular Medicine, Department of Medicine, University College Hospital and Department of Medicine, College of Medicine, University of Ibadan, Nigeria; Division of Cardiovascular Medicine, Department of Medicine, University College Hospital and Department of Medicine, College of Medicine, University of Ibadan, Nigeria; Division of Cardiovascular Medicine, Department of Medicine, University College Hospital and Department of Medicine, College of Medicine, University of Ibadan, Nigeria; Department of Clinical Pharmacology, University College Hospital, Ibadan, Nigeria

## Abstract

**Background:**

Hypertension results in structural and functional changes in the heart. Early detection of abnormalities of cardiac structure and function is important in the assessment and treatment of hypertensive subjects. The aim of this study was to evaluate the utility of the tissue Doppler echocardiographic technique in characterising diastolic and systolic functions in untreated native black African hypertensive subjects.

**Materials and methods:**

Forty consecutive, newly diagnosed, untreated hypertensives with adequate conventional echocardiographic (2-D, M-mode, transmitral and pulmonary Doppler flow velocities) and tissue Doppler echocardiographic images were recruited into the study. The control subjects were apparently normal individuals. Each arm of the study consisted of 21 male and 19 female subjects.

**Results:**

The two groups were comparable by age (48.6 ± 11.35 years in the hypertensives vs 48.1 ± 11.33 years in the controls; *p* = 0.844) and gender distribution (M/F: 21/19 in both groups). Other baseline characteristics, except for blood pressure parameters, which were predictably higher in the hypertensive subjects, were comparable between the two groups. The hypertensive subjects had a lower systolic myocardial velocity (S_m_) and early diastolic myocardial velocity (E_m_) in comparison with the controls (*p* = 0.033 and *p* = 0.018, respectively). The late diastolic myocardial velocity (A_m_) was comparable in the two groups (*p* = 0.430).

**Conclusions:**

Tissue Doppler echocardiography demonstrates diastolic dysfunction relatively early in native African hypertensives and may be useful for detecting subtle deterioration in systolic function.

## Summary

Hypertension has worldwide prevalence^1^ and is a cause of morbidity and mortality because of its effect on the target organs, one of which is the heart. The effects of hypertension are both structural and functional.^2^-^4^ Assessment of the function and/or structure of the heart is an important requisite for intervention and risk stratification in hypertensives. The burden of hypertension is high in blacks and effects on target organs are more severe than in many other races.^5^

Tissue Doppler imaging is a newer echocardiographic imaging technique that is believed to be superior to the older conventional techniques in assessing abnormalities of cardiac function in many conditions affecting the heart. It has also been postulated that it may detect subtle abnormalities in cardiac function.^6^ The tissue Doppler technique is based on the Doppler effect – a change in frequency of ultrasound signals reflected from a moving object – and can be employed to assess regional and global myocardial systolic and diastolic function.

The aim of this study was to assess the utility of the tissue Doppler imaging technique in evaluating left ventricular function in newly diagnosed, untreated native black African hypertensive subjects, among whom there is a paucity of data about the utility of tissue Doppler imaging techniques. Interestingly, this population has a relatively low prevalence of coronary artery disease; hence findings from subjects in this population are less likely to be influenced by sub-clinical coronary artery disease than in many other populations.^7^

## Materials and methods

The study was carried out at the Cardiology Unit in the Department of Medicine of the University College Hospital, Ibadan, Nigeria. Subjects were 80 adult men and women, aged 18 years and older. There were two groups of subjects (matched for age and gender). The first group consisted of 40 consecutive subjects, newly diagnosed with hypertension, with adequate echocardiograms, who met the inclusion criteria. The second group consisted of 40 apparently normal controls.

Hypertension was defined as blood pressure, measured according to standard guidelines, of 140/90 mmHg or higher on at least two occasions.^8^ Subjects were excluded if they had symptoms of cardiac decompensation, valvular heart disease, a history of ischaemic heart disease, or regional wall motion abnormalities on echocardiogram. Other exclusion criteria included co-existing cardiomyopathy, morbid obesity and technically difficult echocardiogram.

Ethical approval was obtained from the institutional ethical review committee and informed consent was obtained from each patient.

Echocardiography was carried out with an ALOKA SSD-1700 echo machine, which was equipped with 2.5–5.0 MHz linear array transducers.

Two-dimensional guided M-mode echocardiography was performed on each subject in the left lateral decubitus position. All measurements were made according to the American Society of Echocardiography leading-edge-to-leading-edge convention.^9^ An average of measurements in three cardiac cycles was taken with simultaneous ECG recordings. All echocardiographic studies were performed by one observer who was blinded to the clinical data of the subjects. In our laboratory, intra-observer concordance correlation coefficients of echocardiographic measurements varied from 0.76 to 0.98.^10^

Left ventricular (LV) volume was estimated using the formula of Teichholz *et al.* and the ejection fraction was calculated using the standard formula.^11^ Fractional shortening was calculated from LV internal dimensions in diastole and systole:

$\frac{LVIDd-LVIDs}{LVIDd}\;\times 100$

where LVIDd is the left ventricular internal diameter in diastole and LVIDs is the left ventricular internal diameter in systole.

Left ventricular mass (LVM) was calculated using the Devereux-modified ASE cube formula:^12^

LVM = 0.8 × (1.04 × (VSTd + PWTd + LVIDd)^3^ – (LVID)^3^ + 0.6

where VSTd is the ventricular septal thickness in diastole and PWTd is the posterior ventricular wall thickness in diastole.

Relative wall thickness (RWT) was calculated as:^13^

$RWT=\;\frac{VSTd+PWTd}{LVIDd}$

Four left ventricular geometric patterns were identified: normal geometry, when indexed left ventricular mass and relative wall thickness were normal; concentric remodelling, when indexed left ventricular mass was normal but relative wall thickness was increased; concentric hypertrophy, when both indexed left ventricular mass and relative wall thickness were increased; eccentric left ventricular hypertrophy, when indexed left ventricular mass was increased but with normal relative wall thickness.^14^ A partition value of 51 g/ht^2.7^ was used since this was the only criterion that demonstrated the optimal threshold value for left ventricular hypertrophy in blacks, irrespective of gender. A partition value of 0.45 was used for the RWT.^14^,^15^

Doppler echocardiography: the mitral inflow velocities were measured from the apical four-chamber view. Measurements included the peak early mitral inflow velocity (E-wave), the peak atrial mitral inflow velocity (A-wave), the E/A ratio, and the deceleration time (DT) (time interval of peak E-wave velocity to its extrapolation to the baseline).

Pulmonary venous flow was obtained using the right upper pulmonary vein in the apical four-chamber view. The measurements obtained were S-wave (systolic forward flow), D-wave (diastolic forward flow) and A-wave (atrial reversal).^16^

Tissue Doppler: pulsed tissue Doppler imaging was performed in the apical four-chamber view. The filter setting was decreased to exclude high-frequency signals, and the gain minimised to allow for clear tissue signals with minimal background noise. The sample volume was positioned as parallel as possible with the lateral mitral annular motion. Variables of the tissue velocity evaluated were peak systolic myocardial velocity (S_m_), early diastolic myocardial velocity (E_m_), late diastolic myocardial velocities (A_m_), the duration of S_m_ and E_m_, A_m_ with isovolumic relaxation time (IVRT), and isovolumic contraction time (IVCT).^17^

## Statistical analysis

Comparison of categorical variables between the groups was by chi-squared test, while continuous variables were compared with the student’s *t*-test for independent groups. Variables potentially related to measures of diastolic function were investigated using Pearson’s correlations. Analysis was performed using SPSS software version 10.0 (SPSS, Inc, Chicago, Illinois).

## Results

Table 1 shows the baseline characteristics of the subjects (hypertensives and normal controls). The two groups were comparable by age, weight, height, body surface area and body mass index. Predictably, however, they differed significantly in blood pressure parameters.

**Table 1:** Baseline Characteristics Of The Subjects

*Characteristics*	*Hypertensives ± SEM (n = 40)*	*Controls ± SEM (n = 40)*	p*-value*
Age (years)	48.6 (11.4)	48.1 (11.3)	0.844
Gender (male/female)	21/19	21/19	1.000
Weight (kg)	70.5 (14.2)	67.3 (12.5)	0.287
Height (cm)	163.3 (9.5)	161.6 (8.8)	0.416
BMI (kg/m^2^)	26.5 (4.98)	25.7 (4.1)	0.474
HR (beats/min)	82.7 (23.34)	72.1 (13.02)	0.015
SBP (mmHg)	153.7 (19.0)	117.7 (13.7)	< 0.0001
DBP (mmHg)	99.6 (11.8)	74.4 (9.3)	< 0.0001
MAP (mmHg)	117.6 (11.9)	88.8 (9.7)	0.002
Pulse pressure (mmHg)	54.1 (18.0)	43.28 (10.9)	< 0.0001

BMI = body mass index; HR = heart rate; SBP = systolic blood pressure; DBP = diastolic blood pressure; MAP = mean arterial blood pressure.

Comparison of the M-mode and conventional Doppler measurements between the two groups (Table 2) revealed that the hypertensives had a thicker septum (*p* < 0.0001), but comparable posterior wall thickness, left ventricular internal diameters, fractional shortening and ejection fraction. Except for atrial mitral inflow velocity (A velocity), which was higher in the hypertensives (*p* = 0.006), all other measured parameters of mitral inflow and pulmonary venous flow were comparable.

**Table 2:** M-Mode And Doppler Echocardiographic Measurements

*Variables*	*Hypertensives ± SEM (n = 40)*	*Controls ± SEM (n = 40)*	p*-value*
IVSd (cm)	1.3 (0.31)	1.0 (0.15)	< 0.0001
IVSs (cm)	1.6 (0.34)	1.5 (0.20)	0.265
PWTd (cm)	0.9 (0.25)	0.9 (0.12)	0.287
PWTs (cm)	2.5 (0.302)	1.4 (0.25)	0.281
LVIDd (cm)	4.6 (0.62)	4.4 (0.66)	0.339
LVIDs (cm)	3.0 (0.69)	2.7 (0.55)	0.071
LVM (g)	184.1 (68.9)	144.9 (41.0)	0.003
LVM/HT^2.7^ (g/m^2.7^)	49.2 (17.65)	39.8 (11.53)	0.006
FS (%)	37.1 (9.18)	38.3 (7.87)	0.534
EF (%)	70.1 (17.52)	75.4 (9.68)	0.098
E (m/s)	0.64 (0.20)	0.63 (0.17)	0.693
A (m/s)	0.65 (0.20)	0.54 (0.15)	0.006
E/A	1.1 (0.57)	1.3 (0.45)	0.191
DT (ms)	185.2 (53.78)	186.8 (47.48)	0.884
S (m/s)	0.46 (0.18)	0.42 (0.13)	0.290
D (m/s)	0.38 (0.14)	0.32 (0.11)	0.137
S/D	1.3 (0.46)	1.4 (0.43)	0.503
PA (m/s)	0.27 (0.09)	0.23 (0.07)	0.062

IVSd = interventricular septum in diastole; IVSs = interventricular septum in systole; PWTd = posterior wall thickness in diastole; PWTs = posterior wall thickness in systole; LVIDd = left ventricular internal diameter in diastole; LVIDs = left ventricular internal diameter in systole; LVM = left ventricular mass; HT = height; FS = fractional shortening; EF = ejection fraction; E = early transmitral diastolic flow velocity; A = late transmitral diastolic flow velocity; DT = deceleration time of the transmitral E-wave; S = pulmonary vein systolic flow velocity; D = pulmonary vein diastolic flow velocity; PA = pulmonary vein reverse flow velocity.

Fifteen (37.5%) of the hypertensive subjects had normal LV geometry, 10 (25%) had concentric remodeling, 11 (27.5%) had concentric hypertrophy, and four (10%) eccentric hypertrophy. The corresponding values for the normal controls were 20 (50%), 14 (35%), 0 (0%) and six (15%), respectively. Hence, 37.5% of the hypertensives had left ventricular hypertrophy compared with 15% in the control group.

Table 3 shows the measured tissue Doppler parameters. The hypertensive subjects had a lower systolic myocardial velocity (S_m_) (0.12 ± 0.03 m/s vs 0.14 ± 0.03 m/s; *p* = 0.033) and early diastolic myocardial velocity (E_m_) (0.14 ± 0.04 m/s vs 0.16 ± 0.05 m/s; *p* = 0.018), but a higher A_m_ duration (91.2 ± 36.97 vs 74.6 ± 16.52; *p* = 0.011). The groups had comparable atrial myocardial velocity (A_m_). The E/E_m_ ratio showed a trend towards a higher value in the hypertensive group. The presence of left ventricular hypertrophy did not result in E/E_m_ that was significantly different from the ratio in subjects without hypertrophy (5.0 ± 1.14 in subjects with LVH vs 4.5 ± 2.20 in subjects without LVH; *p* = 0.243).

**Table 3:** Tissue Doppler Echocardiographic Parameters

*Variables*	*Hypertensives ± SEM (n = 40)*	*Controls ± SEM (n = 40)*	p*-value*
S_m_ (m/s)	0.12 (0.03)	0.14 (0.03)	0.033
E_m_ (m/s)	0.14 (0.04)	0.16 (0.05)	0.018
A_m_ (m/s)	0.13 (0.04)	0.12 (0.03)	0.430
E_m_/A_m_ (m)	1.2 (0.59)	1.4 (0.53)	0.109
S_m_D (ms)	218.9 (63.28)	237.2 (32.66)	0.108
E_m_D (ms)	113.8 (60.41)	11.1 (26.367)	0.805
A_m_D (ms)	91.2 (36.97)	74.6 (16.52)	0.011
E/E_m_	5.0 (1.92)	4.2 (2.00)	0.054
IVRT (ms)	126.3 (48.47)	137.0 (40.02)	0.284
IVCT (ms)	102.8 (30.18)	111.0 (25.51)	0.197

S_m_ = peak systolic myocardial velocity; E_m_ = early diastolic myocardial velocity; A_m_ = late diastolic myocardial velocities; S_m_D = duration of S_m_; E_m_D = duration of E_m_; A_m_D = duration of A_m_; IVRT = isovolumic relaxation time; IVCT: isovolumic contraction time.

The early diastolic myocardial velocity (E_m_) was inversely related to the diastolic and systolic blood pressures, the septal and posterior wall diameters in diastole, and the E/E_m_ ratio, and directly related to S_m_.

Table 4

**Table 4:** Correlates Of Early Diastolic Myocardial Velocity

*Variables*	*Correlation coefficient*	p*-value*
DBP	–0.374	0.01
SBP	–0.363	0.01
PP	–0.172	0.126
LVIDd	–0.214	0.056
IVSd	–0.228	0.042
PWTd	–0.270	0.015
LVM	–0.366	0.009
LVM/HT^2.7^	–0.421	< 0.0001
Sm	0.534	< 0.0001
E/E_m_	–0.620	< 0.0001

DBP = diastolic blood pressure; SBP = systolic blood pressure; LVIDd = left ventricular internal diameter in diastole; IVSd = interventricular septum in diastole; PWTd = posterior wall thickness in diastole; LVM = left ventricular mass; HT = height; S_m_ = peak systolic myocardial velocity; E = early transmitral diastolic flow velocity; E_m_ = early diastolic myocardial velocity.

## Discussion

This study shows that in native African hypertensives with preserved systolic function and only mild diastolic dysfunction and mild structural abnormalities by conventional echocardiographic technique, the tissue Doppler technique is able to demonstrate not only abnormalities of left ventricular filling but also some deterioration in systolic function, in comparison with apparently normal subjects.

Increased left ventricular septal wall thickness and left ventricular mass, found in the hypertensives in this study when compared with the controls, are some of the changes in left ventricular structure and geometry that may accompany hypertension. Similar findings have been documented in previous studies in different population groups.^18^-^21^

The finding of a significantly lower peak systolic myocardial velocity S_m_ (known to correlate with LV ejection fraction^22^) in the hypertensives in this study, despite having indices of systolic function comparable with the controls by conventional echocardiographic technique, may be a reflection of the ability of the tissue Doppler technique to detect even subtle systolic abnormalities. This is similar to the findings by Kobayashi *et al.* in untreated hypertensives.^23^

Poulsen *et al.*^24^ demonstrated that a lower systolic myocardial velocity in hypertensives is even more prominent in subjects with demonstrable diastolic dysfunction. It is well known that LV systolic function, assessed using the ejection fraction, is load dependent and may not reflect the true contractile state of the myocardium. On the other hand, tissue Doppler velocities are known to be load independent.^25^,^26^

Furthermore, tissue Doppler imaging is useful for easy and reproducible distinction of ‘pseudonormalisation’ of LV diastolic function from the ‘normal’ pattern. The Doppler indices of transmitral flow change from the impaired relaxation pattern, in which there is a reduction in the amplitude but an increase in the duration of the E-velocity, to the pseudonormalised pattern with progressive elevation of the LA pressure. The latter pattern would require the demonstration of a reversal of the S/D ratio and a prolongation and increased amplitude of the atrial reversal wave, by the cumbersome pulse-wave Doppler analysis of the pulmonary venous flow, to distinguish it from the normal pattern.^27^

There was a trend towards a lower ejection fraction in the hypertensive group. This finding is contrary to the expected accentuation of LV systolic function early in hypertension. This trend, however, has been consistent in earlier studies in our centre in newly diagnosed hypertensives and also in hypertensives with and without ECG strain pattern.^28^

In a study comparing echocardiographic parameters in whites and blacks (African-Americans) in which the majority of the study population was untreated, the LV ejection fraction was higher in the black study group. This difference may be indicative of the fact that our study population may have been more heterogeneous with regard to the duration of hypertension, with the unifying factor being the absence of previous pharmacological interventions and the absence of symptoms of cardiac decompensation. It may, however, be reinforcing the speculation that findings in African-American populations or Africans in diasporas may not necessarily be applicable to native African populations.

The probable heterogeneity of our study population is further emphasised by the significantly higher left ventricular mass and wall thickness in the hypertensive subjects compared with that of the control subjects. Lower peak early diastolic myocardial velocity (E_m_) in the hypertensives is a pointer to abnormality of diastolic filling in the subjects with hypertension. E_m_ is known to be the most striking tissue Doppler index of impaired relaxation. 29,30 It has been shown to correlate well with parameters of myocardial relaxation and it is sensitive to changes in the structure and neurohormonal mechanisms of the myocardium.^31^

Similar findings were documented by Almeida and co-workers^32^ in a subset of young hypertensive adults with autosomaldominant polycystic kidney disease (APKD), who had a higher LV mass index, decreased systolic peak velocity S_m_ wave, and lower tissue Doppler index peak early diastolic annular velocity E_m_ wave, compared with normotensive APKD subjects and the normal controls. Peterson *et al.* also demonstrated lower S_m_ and E_m_ in obese but otherwise healthy women. Von Bibra *et al.*^33^ found that tissue Doppler imaging may demonstrate abnormal systolic and diastolic function in diabetics even when traditional echocardiographic indices of systolic myocardial function are still normal. Furthermore, lower S_m_ and E_m_ have been suggested as possible predictors of mortality in hypertensives.^34^ The significantly increased A_m_ duration in the hypertensives in this study was probably due to the higher LV wall thickness in this group, since A_m_ has been shown to correlate with wall thickness.^35^

The E/E_m_ ratio showed a trend towards a higher value in the hypertensive group. This may have been due to diastolic dysfunction in these hypertensives. The E/E_m_ ratio related well to pulmonary capillary wedge pressure measured by invasive means in other studies.^17^,^36^ It is not surprising that this difference did not reach statistical significance, considering the inclusion characteristics of the study groups.

The relationships between E_m_ as an index of diastolic function and measures of left ventricular hypertrophy and geometry, and blood pressure parameters is in keeping with the expected relationships with diastolic function. It is well known that diastolic dysfunction occurs not only as a result of left ventricular hypertrophy but also as a result of fibrosis, which is expected to accompany pathological hypertrophy. High blood pressure and diastolic dysfunction are linked via alteration of the collagen framework, which occurs in hypertension, leading to increased left ventricular stiffness and impaired left ventricular filling.^37^ Fibroblast activation in this case occurs through humoral or mechanical means. Consequently, diastolic dysfunction, detectable by tissue Doppler imaging, may occur even before overt hypertrophy. It is has also been demonstrated that deterioration in systolic and diastolic function occurs simultaneously.^24^

## Limitations of the study

No history suggesting angina and an electrocardiogram without features of ischaemia may not be sufficient to completely exclude coronary artery disease. This would require angiography, which was not carried out in these subjects; hence our results could have been influenced by sub-clinical coronary artery disease. Regional function by tissue Doppler would have further helped to characterise left ventricular function. This was not included in our study.

The strict inclusion criteria in this study imply that the results may not be applicable over a wide range of cardiovascular diseases. A study with a much larger sample size would be helpful in further characterising left ventricular function in hypertensives and subjects with other cardiovascular diseases within this racial group. Multiple comparisons of the different LV geometry in relation to tissue Doppler parameters were also not possible because of the small sample size in this study.

## Conclusions

Using tissue Doppler techniques, deterioration of left ventricular systolic function is demonstrable relatively early in the natural history of hypertensive heart disease in native African hypertensives when compared with the function in apparently normal subjects. This is in addition to its demonstration of the well-recognised abnormalities of left ventricular filling (diastolic dysfunction) and left ventricular structure.
